# Synthetic MRI in the detection and quantitative evaluation of sacroiliac joint lesions in axial spondyloarthritis

**DOI:** 10.3389/fimmu.2022.1000314

**Published:** 2022-09-26

**Authors:** Ke Zhang, Chaoran Liu, Yunfei Zhu, Wenjuan Li, Ximeng Li, Jing Zheng, Guobin Hong

**Affiliations:** ^1^ Department of Radiology, the Fifth Affiliated Hospital of Sun Yat-sen University, Zhuhai, China,; ^2^ Department of Rheumatology and Immunology, the Fifth Affiliated Hospital of Sun Yat-sen University, Zhuhai, China

**Keywords:** axial spondyloarthritis, synthetic MRI, bone marrow edema, quantitative mapping, fat metaplasia

## Abstract

**Objective:**

Our primary objective was to verify the hypothesis that synthetic magnetic resonance imaging (MRI) is similar to conventional MRI in detecting sacroiliac joint lesions in patients with axial spondyloarthritis (axSpA). A secondary objective was to assess the quantitative value of synthetic mapping in bone marrow edema (BME) and fat metaplasia.

**Methods:**

A total of 132 axSpA patients who underwent synthetic and conventional MRI from October 2019 to March 2021 were included in this prospective study. Two independent readers visually evaluated active inflammatory (BME, capsulitis, enthesitis, and inflammation at site of erosion) and structural lesions (erosion, sclerosis, ankylosis, and fat metaplasia) of the sacroiliac joints on conventional and synthetic magnetic resonance (MR) images. In addition, T1, T2, and proton density (PD) values, which were generated by synthetic mapping, were used to further quantitatively evaluate BME and fat metaplasia. A McNemar test was used to compare the differences between the two methods in the detection of sacroiliac joint lesions. Intraclass correlation coefficients (ICCs) were used to assess the inter-reader consistency of quantitative values. Mann–Whitney tests were performed, and receiver operating characteristic (ROC) curves were created for all quantitative analyses.

**Results:**

There were no statistical difference between synthetic and conventional MRI in the detection of sacroiliac joint lesions (all *p*-values *>* 0.05). A total of 103 images of BME and 111 images of fat metaplasia were quantitatively evaluated using T1, T2, and PD values. The consistency of quantitative values among readers was good (ICC 0.903–0.970). T1 and T2 values were consistently higher in BME than in normal marrow (*p* < 0.001), but PD values were not significantly different (*p* = 0.830). T2 and PD values were higher in fat metaplasia than in normal marrow, but T1 values were lower (*p <* 0.001). In the case of BME, T1 values had greater diagnostic efficiency [area under the curve (AUC) 0.99] than T2 values (AUC 0.78). There were no significant differences in the diagnostic efficiency of T1 (AUC 0.88), T2 (AUC 0.88), and PD (AUC 0.88) values in the case of fat metaplasia.

**Conclusion:**

Synthetic MRI is as effective as conventional MRI in detecting sacroiliac joint lesions in patients with axSpA. Furthermore, synthetic mapping can accurately quantify BME and fat metaplasia.

## Introduction

Axial spondyloarthritis (axSpA) is a type of chronic inflammatory arthritis that mainly affects the sacroiliac joint and spine, causing inflammatory low back pain, and even activity limitation (1–3). In axSpA patients with sacroiliitis, imaging shows both active inflammatory and structural lesions. Magnetic resonance imaging (MRI) can specifically detect inflammatory lesions that cannot be detected by conventional radiography or computed tomography (CT), and is superior to radiography for the detection of structural lesions (4, 5). In addition, MRI involves no radiation exposure and is suitable for following up patients (6). Therefore, MRI is recommended as the preferred imaging method for the diagnosis of axSpA (7). However, conventional MRI can evaluate sacroiliac joint lesions only qualitatively or semiquantitatively, and cannot provide accurate, repeatable, or objective quantitative values that can provide more information about axSpA.

Recent studies have shown that dynamic contrast material-enhanced MRI (DCE-MRI), conventional T2 mapping, diffusion-weighted imaging (DWI), and the dual-energy CT (DECT) virtual non-calcium technique have quantitative diagnostic value for axSpA. DCE-MRI can assess inflammatory lesions and further evaluate the curative effect of treatments received by axSpA patients by generating hemodynamic parameters. However, the use of contrast agents has limitations and is associated with side effects (8). DWI, by enabling measurement of the apparent diffusion coefficient (ADC), can quantify inflammatory lesions. However, as the size of the lesion is related to the DWI b-value, measurement is biased and inter-observer consistency is poor (9–12). T2 values, which reflect collagen content, tissue anisotropy, and the presence of water molecules, can be used to assess bone marrow and cartilage lesions but the consistency and repeatability of T2 values are poor (13–15). At present, these techniques can quantitatively assess only inflammatory lesions and cannot be used in clinical diagnosis alone, without conventional MRI. DECT is of some value in the detection and quantitative evaluation of bone marrow edema (BME), but is still not widely available (16).

Magnetic resonance image compilation (MAGiC), as one form of synthetic MRI, can perform arbitrary contrast imaging and multi-quantitative mapping in a single scan. MAGiC sequencing based on multiple delay multiple echo (MDME) can provide an indication of inherent tissue properties by measuring relaxation time (longitudinal, T1, relaxation, transverse, T2, relaxation) and proton density (PD) (17–20). Synthetic MRI has been widely used to scan the brain (21–23). In the musculoskeletal system, synthetic MRI has in recent years been successfully used to scan knees and the spine (24–30). Our previous research showed that synthetic MRI could provide imaging quality similar to that of conventional MRI contrast images of the sacroiliac joints (31).

In this study, we aimed to determine if synthetic MRI can detect sacroiliac joint lesions associated with axSpA and could replace conventional MRI. We also aimed to assess the performance of the quantitative mapping generated by synthetic MRI for the diagnosis of BME and fat metaplasia.

## Materials and methods

### Study participants

**Figure 1:**
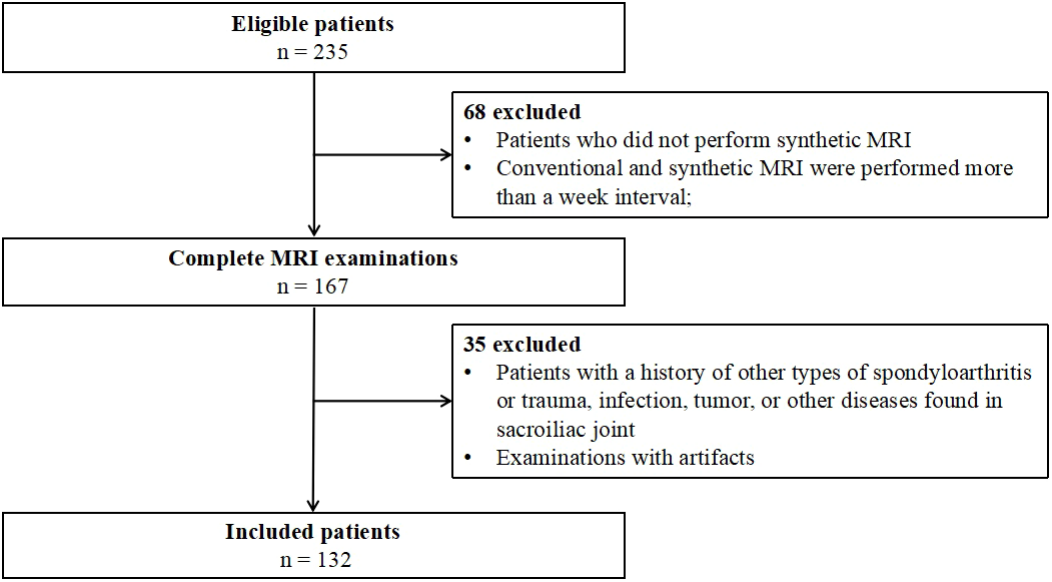
Flow diagram of study participants.

This prospective study was approved by the Ethics Committee of the Fifth Affiliated Hospital of Sun Yat-sen University, and informed consent was obtained from all patients. A total of 235 patients diagnosed with axSpA by rheumatologists were recruited from October 2019 to March 2021. The diagnostic criteria for axSpA are back pain of at least 3 months’ duration, age at onset < 45 years, and sacroiliitis on imaging plus at least one feature of SpA or the presence of human leukocyte antigen B27 (HLA-B27) plus two or more SpA features. SpA features include arthritis, uveitis, dactylitis, good response to non-steroidal anti-inflammatory drugs (NSAIDs), and elevated levels of C-reactive protein (CRP) (3). We excluded (1) patients who did not undergo synthetic MRI, or in whom the interval between synthetic and conventional MRI was more than 1 week; (2) patients with tumors, trauma, infection, or other lesions in the sacroiliac joints; and (3) patients whose images showed artifacts that precluded further evaluation. The final number of patients included in the study was 132 (**Figure 1**).

### MRI protocol

All patients underwent sacroiliac joint MRI examination on a 3.0-T scanner (Signa™ Pioneer, GE Healthcare, Chicago, IL, USA) using a 16-channel abdomen phased-array coil. MRI examination included synthetic and conventional sequences. **Table 1** shows detailed scanning parameters.

**Table 1:** Magnetic resonance imaging (MRI) protocol.

Parameter	Conventional MRI		Synthetic MRI(MAGiC)
	T1WI	STIR	
TR (ms)	602	5856	4521
TE (ms)	15.2~20.3	28	23.5
FOV (mm^2^)	240 × 240	240 × 240	240 × 240
Matrix	320 × 256	320 × 256	320 × 256
Thickness/gap (mm)	4/1	4/1	4/1
Echo train length	16	16	16
Number of excitations	2	2	2
Scanning time	2 min 32 s	4 min 24 s	6 min 08 s

FOV, field of view; STIR, short time of inversion recovery; TE, echo time; TR, repetition time.

### Visual image analysis

Conventional and synthetic MR images were analyzed by two readers (one with 9 years’ and one with 2 years’ experience in musculoskeletal imaging diagnosis), with a 1-month interval between the two readings. The readers worked independently and examined the images, in random order, for sacroiliac joint lesions. In the case of disagreement, the results were determined by a third reader with 25 years’ experience of musculoskeletal imaging diagnosis. All readers were blind to clinical characteristics and laboratory indicators and trained in the 2019 assessment of the Spondyloarthritis International Society (ASAS)’s updated definitions of sacroiliac joint MRI lesions in spondyloarthritis (5).

In accordance with the 2019 ASAS updated definitions, sacroiliac joint lesions in MRI can be divided into active (BME, capsulitis, enthesitis, and inflammation at site of erosion) and structural lesions (sclerosis, ankylosis, erosion, and fat metaplasia) (5). Erosion shows a loss of signal in cortical bones and adjacent marrow on T1-weighted images (T1WI) sequences, and sclerosis appears as a very low signal on all sequences. Fat metaplasia shows a homogeneously higher signal than normal bone marrow on T1WI sequences and a sharp border in the subchondral area. Ankylosis shows continuity between the ilium and the sacrum, with a similar signal to bone marrow on the T1WI sequence. BME shows subchondral hyperintensity on the short time of inversion recovery (STIR) sequence. Capsulitis shows increased signal on the STIR sequence, which is observed at the cranial or caudal areas on oblique coronal images. Enthesitis shows high signal in bone marrow or soft tissue at the point of attachment of tendons and ligaments on the STIR sequence. Inflammation at the site of erosion shows increased signal on the STIR sequence at the site of erosion. BME, fat metaplasia, and sclerosis were considered severe if they involved more than half of the articular surface of the sacroiliac joints, and mild if they involved less than half. Erosion may be small and discrete or large, causing pseudo-widening of the joint. Ankylosis was categorized as absent or as involving the joint partially or completely. The left and right sacroiliac joints of each patient were evaluated separately and, thus, a total of 264 sacroiliac joints were evaluated.

### Quantitative analysis

Two readers (one with 9 years’ and one with 12 years’ experience, as reported above) determined the regions of interest (ROIs) of BME and fat metaplasia based on the final qualitative results. Areas of BME and fat metaplasia identified on both synthetic and conventional MR images were measured on the quantitative mapping generated by synthetic MRI to obtain T1, T2, and PD values. The mean value was calculated based on each sacroiliac joint with a lesion. Necrosis, blood vessels, and cystic regions were to be avoided when determining ROIs. In addition, quantitative values for normal bone marrow surrounding each lesion were measured and the mean calculated. The final quantitative values for lesions and normal marrow of sacroiliac joints were the average of the values determined by the two readers.

### Statistical analysis

The data were analyzed by IBM SPSS Statistics Version 26.0 (IBM Statistics, Armonk, NY, USA). Continuous variables with a normal distribution are presented as mean ± SD; non-normal variables are reported as median (minimum, maximum). A McNemar test was used to compare the differences between the two methods in the detection of sacroiliac joint lesions. Intraclass correlation coefficients (ICCs) were used to assess the inter-reader consistency of quantitative values. It is generally considered that an ICC below 0.4 indicates poor reliability, whereas an ICC greater than 0.75 indicates good reliability. Mann–Whitney tests were used to compare quantitative values among normal bone marrow and lesions. Receiver operating characteristic (ROC) curve analysis and the area under the curve (AUC) were used to confirm the quantitative cut-off values for lesions (BME and fat metaplasia); the corresponding sensitivity, specificity, and accuracy were also calculated. Differences were considered statistically significant at *p*-values < 0.001.

## Results

A total of 132 participants (aged 18–58 years), comprising 98 men (aged 18–55 years) and 34 women (aged 19–58 years), were enrolled in this study, of whom 103 (78%) were HLA-B27 positive.

### Visual image analysis

The McNemar test results showed that there were no statistically significant differences between synthetic and conventional MRI in the detection of sacroiliac joint lesions (all *p* > 0.05). The detailed data are shown in **Table 2**.

**Table 2:** Assessment of sacroiliac joint lesions with conventional and synthetic MRI.

Sacroiliac joint lesions	Conventional MRI	Synthetic MRI	*P*
**Structural changes**			
**Erosion**			
Absent	71 (27)	66 (25)	
Small	153 (58)	154 (58)	0.311
Large	40 (15)	44 (17)	
**Fat metaplasia**			
Absent	153 (58)	148 (56)	
Mild	82 (31)	85 (32)	0.479
Severe	29 (11)	31 (12)	
**Sclerosis**			
Absent	117 (44)	110 (42)	
Mild	114 (43)	119 (45)	0.170
Severe	33 (13)	35 (13)	
**Ankylosis**			
Absent	169 (64)	167 (63)	
Partially involved	72 (27)	74 (28)	0.779
All involved	23 (9)	23 (9)	
**Active changes**			
**BME**			
Absent	161 (61)	156 (59)	
Mild	75 (28)	80 (30)	0.096
Severe	28 (11)	28 (11)	
**Capsulitis**			
Absent	248 (94)	246 (93)	0.687
Present	16 (6)	18 (7)	
**Enthesitis**			
Absent	217 (82)	214 (81)	0.508
Present	47 (18)	50 (19)	
**Inflammation at** **site of erosion**			
Absent	212 (80)	206 (78)	0.180
Present	52 (20)	58 (22)	

Data in parentheses are percentages.

### Quantitative analysis

A total of 103 images of BME and 111 images of fat metaplasia were quantitatively analyzed. The consistency of quantitative values among readers was good in all cases, as shown in **Table 3** (ICC 0.903–0.970).

**Table 3:** Inter-reader agreement (mean difference and, in parentheses, 95% confidence intervals) of T1, T2, and PD values.

	BME	Normal marrow	Fat metaplasia	Normal marrow
T1 value	0.942 (0.916 to 0.961)	0.938 (0.904 to 0.960)	0.952 (0.931 to 0.967)	0.970 (0.955 to 0.980)
T2 value	0.949 (0.926 to 0.966)	0.951 (0.928 to 0.966)	0.965 (0.950 to 0.976)	0.947 (0.915 to 0.966)
PD value	0.945 (0.920 to 0.963)	0.930 (0.896 to 0.952)	0.906 (0.865 to 0.935)	0.903 (0.807 to 0.966)

BME, bone marrow edema; PD, proton density.

**Figure 2:**
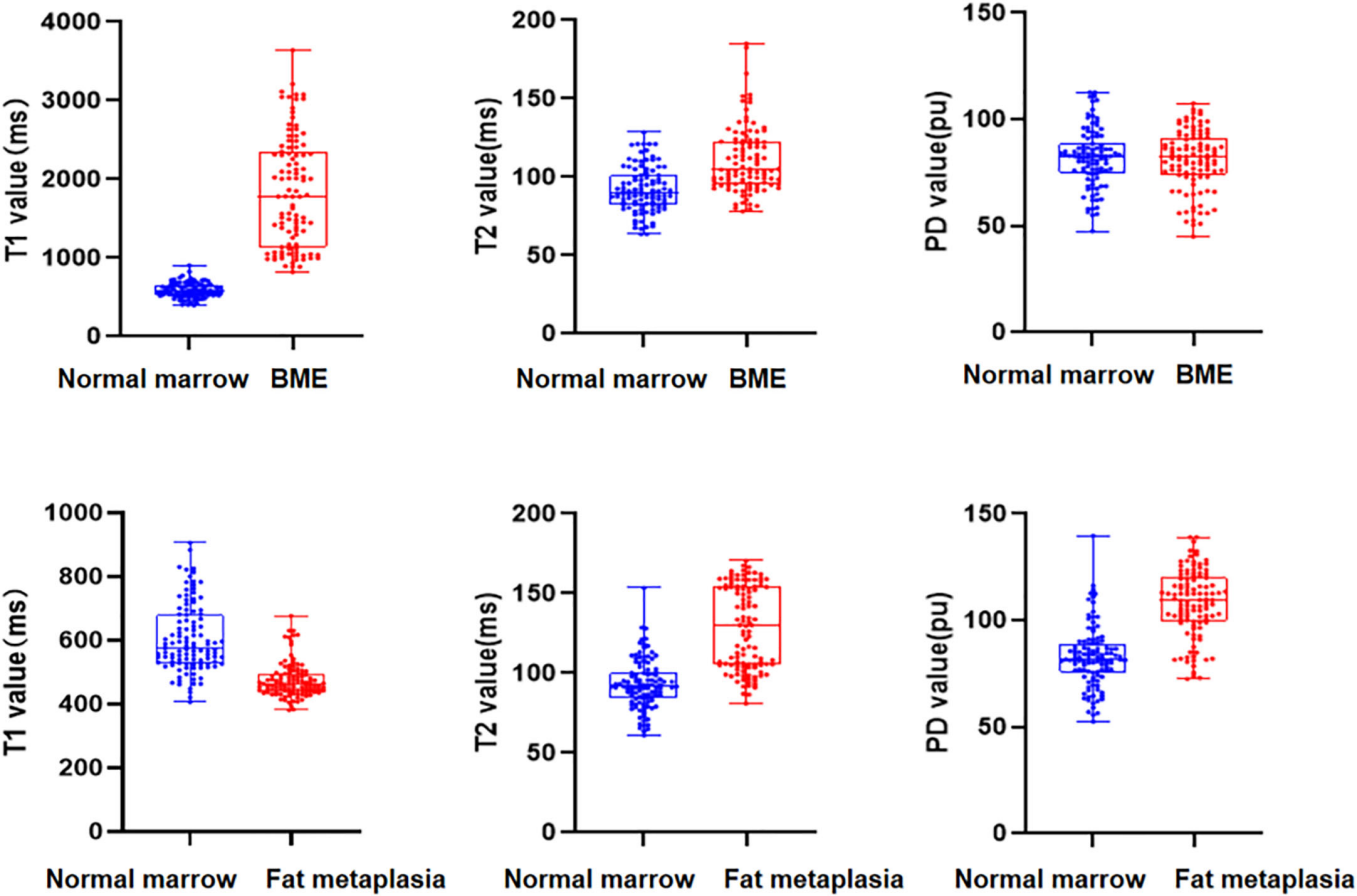
Box scatterplots show T1, T2, and proton density (PD) values in normal marrow and bone marrow edema (BME)/fat metaplasia of the sacroiliac joint in axial spondyloarthritis (axSpA) patients.

Quantitative values for lesions and corresponding normal marrow obtained by quantitative mapping are shown in **Table 4** and **Figure 2**. We found that T1 and T2 values were always higher in BME than in normal marrow (*p* < 0.001). However, there was no significant difference in PD values between BME and normal marrow (*p* = 0.830). T2 and PD values were consistently higher in fat metaplasia than in normal marrow, but the T1 value was lower (*p* < 0.001).

**Table 4:** T1, T2, PD values of BME, fat metaplasia, and normal marrow.

	BME	Normal marrow	*Z*	*p-*value	Fat metaplasia	Normal marrow	*Z*	*p-*value
T1 value (ms)	1780.5 (823, 3640.5)*	586.2 ± 94.1	–12.388	< 0.001	474.3 ± 55.3	575 (406.5, 906)*	–9.882	< 0.001
T2 value (ms)	104.7 (78.3, 185)*	91.8 ± 14.3	–6.936	< 0.001	129.5 (81, 170.5)*	92.8 ± 15.4	–9.673	< 0.001
PD value (pu)	82.1 ± 13.6	82.5 (45.0, 107.5)*	–0.215	0.830	109.5 (72.5, 139.1)^*^	82.7 ± 14.3	–9.667	< 0.001

*Data are median (minimum, maximum). Remaining values are mean ± SD. BME, bone marrow edema; PD, proton density; pu, percentage unit.

The p-value was < 0.001 in the comparison of the T1 and T2 values among BME and normal marrow.

The p-value was < 0.001 in the comparison of the T1, T2, and PD values among fat metaplasia and normal marrow.

**Figure 3:**
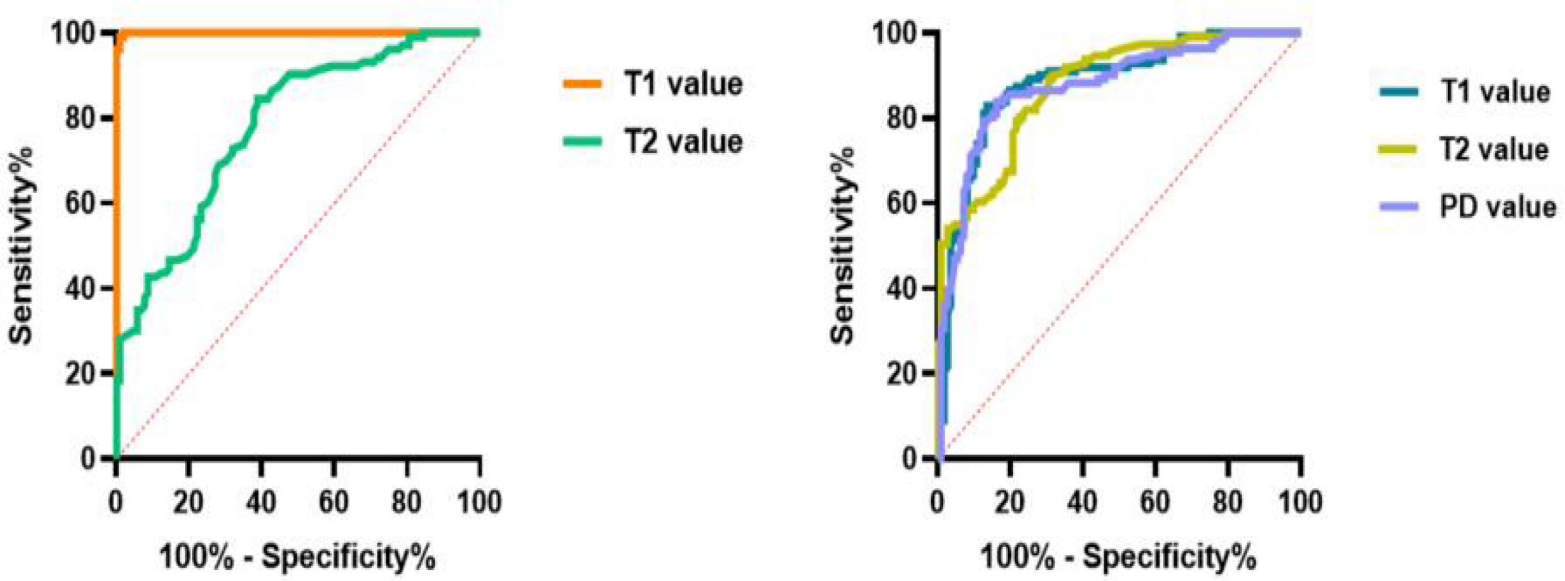
Graphs show the receiver operating characteristic curves calculated from T1, T2, and proton density (PD) values in detecting bone marrow edema (BME) and fat metaplasia.

The ROC curve analysis of quantitative values for BME and fat metaplasia is shown in **Figure 3** and **Table 5**. We found that, in the case of BME, T1 values had greater diagnostic efficacy than T2 values. However, there were no significant differences between the efficacy of T1, T2, and PD values for the diagnosis of fat metaplasia.

**Table 5:** Diagnostic performance of synthetic magnetic resonance imaging in quantitative analysis of BME and fat metaplasia.

Parameter	BME		Fat metaplasia		
	T1 value (ms)	T2 value (ms)	T1 value (ms)	T2 value (ms)	PD value (pu)
Sensitivity (%)	100	84.5	82.9	89.2	83.8
Specificity (%)	98.1	61.2	86.5	69.4	83.8
Accuracy (%)	99	72.8	84.7	79.3	83.8
AUC	0.99	0.78	0.88	0.88	0.88
Cut-off value	798.3	92.65	511	97	92.5
*p-*value	<0.001	<0.001	<0.001	<0.001	<0.001

AUC, area under the curve; BME, bone marrow edema; PD, proton density.

## Discussion

At present, the diagnosis of axSpA-related sacroiliac joint lesions based on imaging findings largely relies on subjective qualitative or semiquantitative methods. Recent studies have shown that conventional T2 mapping, DWI, and DCE technology have quantitative diagnostic value for axSpA, but also have limitations. In this study, we assessed the potential of synthetic MRI as a new quantitative imaging method for the detection and quantitative evaluation of sacroiliac joint lesions. The advantage of synthetic MRI over other technologies is that it can simultaneously acquire multiple quantitative mapping and arbitrary contrast images in a single scan. In addition, the time required is short, so the technique is suitable for axSpA patients who experience lower back pain at rest.

**Figure 4:**
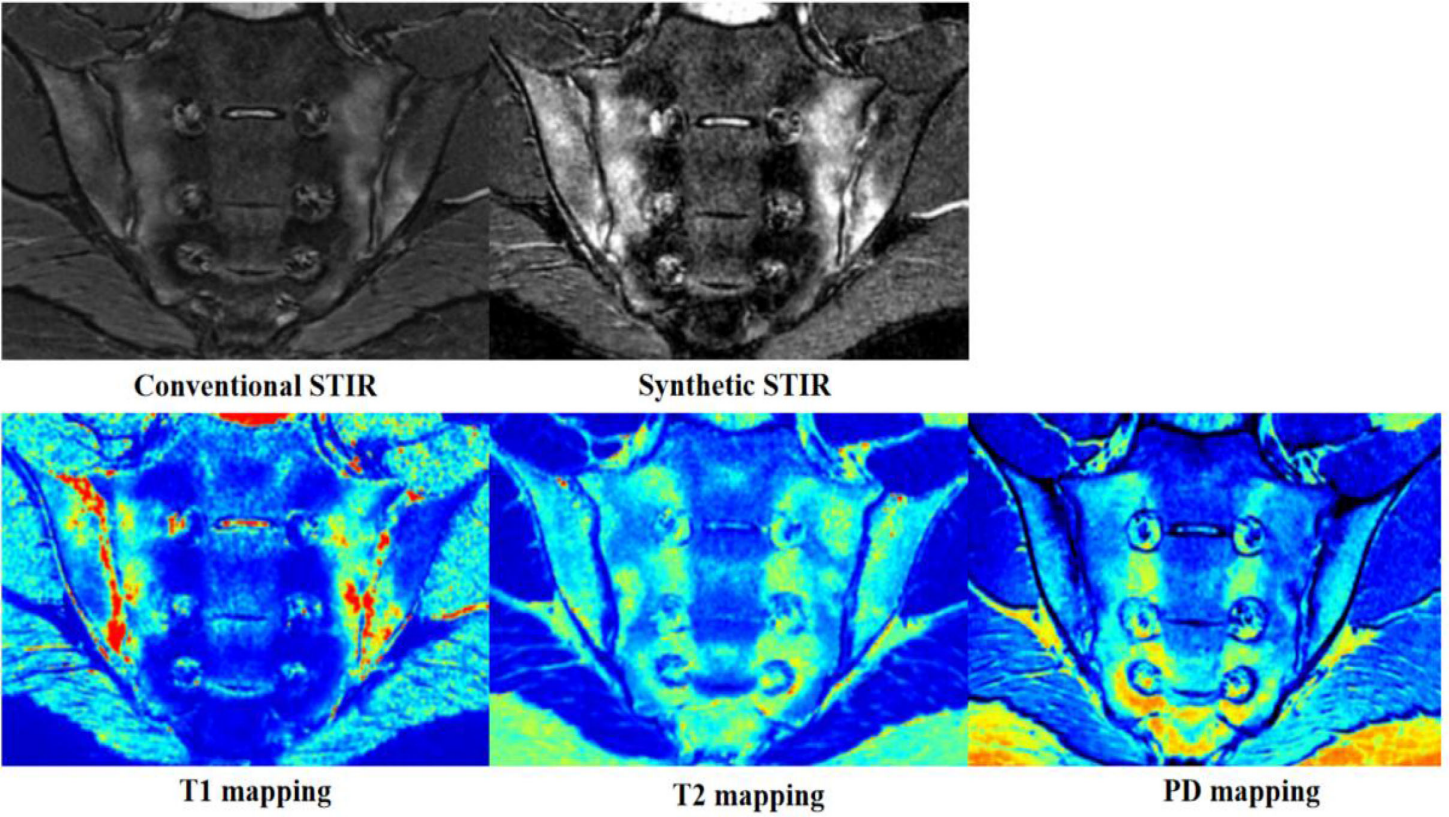
Images from a 19-year-old man with axial spondyloarthritis (axSpA) who presented with a 1-year history of inflammatory back pain and who was human leukocyte antigen B27 (HLA-B27) positive. Conventional and synthetic short time of inversion recovery (STIR) images show severe bone marrow edema (BME) in the bilateral sacroiliac joints. The corresponding areas are green, yellow, and red on T1 mapping; green-yellow on T2 mapping; and blue and wathet blue on proton density (PD) mapping.

Our results showed that there were no differences in the detection of sacroiliac joint lesions between the two methods. In contrast to conventional MRI, with synthetic MRI, contrast images are obtained by adjusting repetition time (TR), echo time (TE), and the reversal time after quantitative mapping, facilitating diagnosis (18). In addition, the reconstructed contrast images of synthetic MRI show the same layer in different sequences, and there is no positioning bias due to scanning time, which is helpful in the accurate localization of lesions. We found that STIR fat suppression was better with the QRAPMASTER method than with conventional MRI (**Figure 4**). This is similar to the results of a study that used synthetic MRI on the knee (25). We believe that the improved STIR fat suppression of synthetic MRI is in part the result of B1 inhomogeneity correction with the use of local effective flip angles. We found that some lesions were clearer and exhibited greater contrast with normal bone marrow on synthetic MR images than on conventional MR images. Consistency among readers was also greater in the case of MR images. In future studies we will use CT as the reference standard with which to compare the detection efficiency of structural lesions by synthetic and conventional MRI.

**Figure 5:**
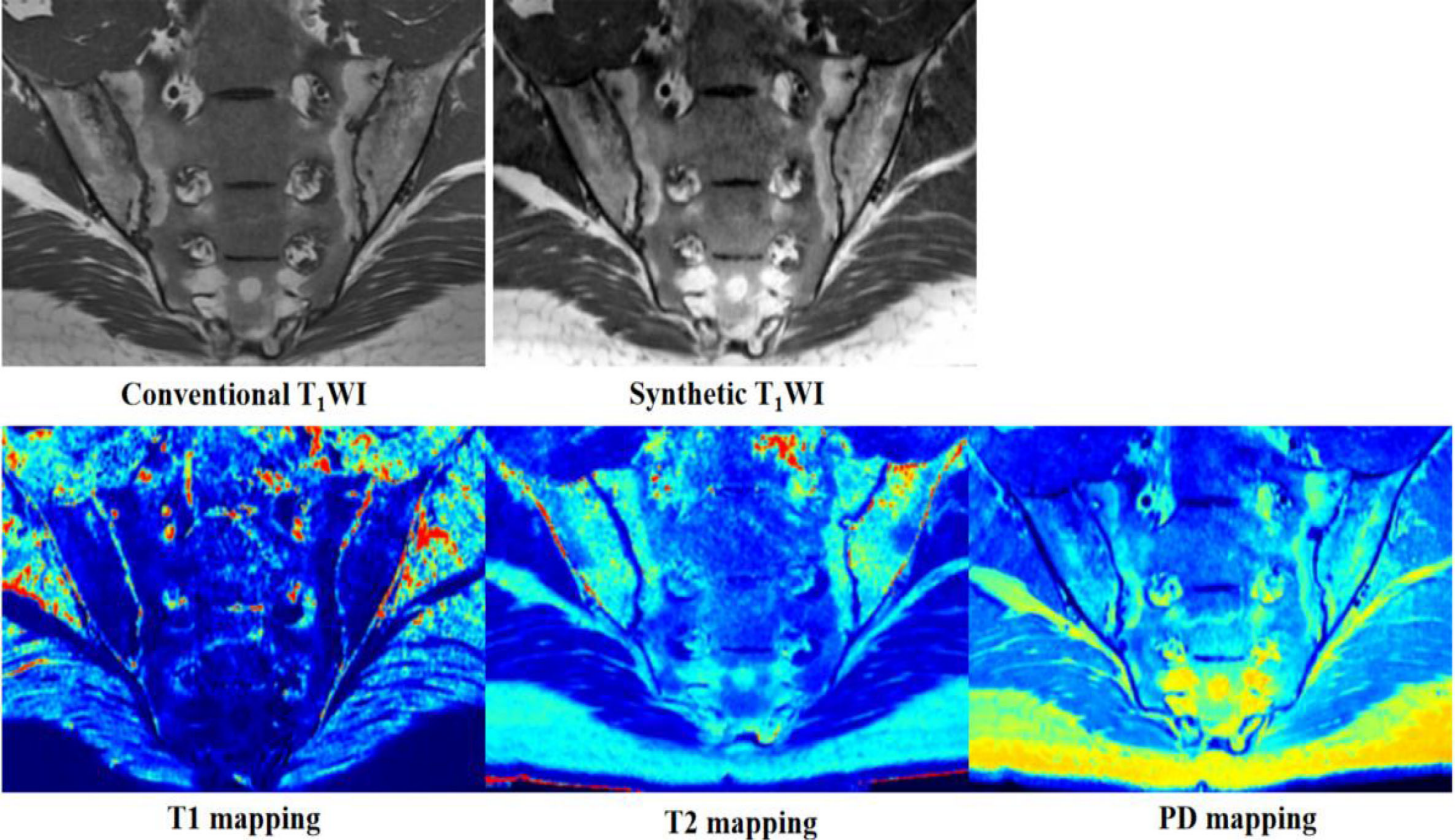
Images from a 29-year-old man with axial spondyloarthritis (axSpA) who presented with a 5-year history of inflammatory back pain and who was human leukocyte antigen B27 (HLA-B27) positive. Conventional and synthetic T1WI images show severe fat metaplasia in the bilateral sacroiliac joints. The corresponding areas are blue on T1 mapping; green, yellow, and red on T2 mapping; and wathet blue or green-yellow on proton density (PD) mapping.

Quantitative analysis showed that both T1 and T2 values are helpful in the diagnosis of BME of the sacroiliac joints, as both values were consistently higher in BME than in normal marrow. However, T1 values performed better than T2 values. This is because T1 values reflect changes in free water content, whereas T2 values are sensitive to tissue water content and the motion of water molecules. BME leads to increased water content and vascular leakage, resulting in an increase in T1 and T2 values (32, 33). Quantitative mapping of MR images is based on analysis of colors, which can be described as cool (such as blue and green) or warm (such as yellow and red). Warm colors indicate higher values. On quantitative mapping, normal bone marrow typically appears blue or blue-green. The color of BME ranged from wathet blue to red (**Figure 4**), which is similar to the findings of our previous study (34, 35). PD values are largely unhelpful in the identification of BME because of the large variation in PD values in normal bone marrow. T1, T2, and PD values are similarly helpful in the diagnosis of fat metaplasia. T2 and PD values were higher in fat metaplasia than in normal marrow, whereas T1 values were significantly lower in fat metaplasia than in normal marrow. In addition, fat metaplasia could be identified by color, which ranged from wathet blue to red (**Figure 5**). An increase in PD values may be due to the high proton density of lipid molecules. Lipids are medium-sized molecules, and their motion frequency is similar to the Larmor frequency, so T1 TR is shortened and T2 TR is longer. We also found that, in some cases, quantitative values were not positively correlated with the level of signal on conventional images. We have posited that the quantitative value of synthetic MRI may be more likely to reflect pathophysiological changes due to axSpA, but further studies will be necessary to verify this hypothesis. Our preliminary results provided T1, T2, and PD cut-off values for fat metaplasia and BME, and as a result we believe that quantitative mapping of images generated by synthetic MRI has the potential to act as a quantitative index, that is as a measure of the severity of disease and pathophysiological changes.

Our work has some limitations. First, our initial quantitative assessment of BME and fat metaplasia was based on areas of bone marrow chosen for their ease of localization and measurement. However, dynamic changes in lesions caused by complex pathophysiology processes in axSpA provide more information about treatment and prognosis; for example, the development of fat metaplasia following BME is thought to be associated with a poor prognosis. Therefore, further longitudinal quantitative assessment of lesions in the future will provide more information about axSpA. Second, we did not obtain axial synthetic MR images; instead we assessed sacroiliac lesions on the basis of only oblique coronal conventional and synthetic MR images. Although, for the observation of lesions, the oblique coronal plane is the most important plane, to achieve optimum sensitivity; both images taken in the oblique coronal plane and axial planes images are needed. In the future, when using synthetic MRI, we will image sacroiliac joints in the axial plane to facilitate diagnosis. Third, our research did not explore the correlation between quantitative values and clinical features or laboratory indicators, which would be helpful for the application of synthetic MRI in clinical work in the future. Fourth, since there is no gold standard for the diagnosis of sacroiliac joint lesions, it remains uncertain whether synthetic or conventional MRI, both of which produce inconsistent results, is more accurate. However, CT is currently considered the reference standard for the visualization of some structural lesions. Therefore, we will in the future use CT as the reference standard to compare the efficiency of detection of structural lesions using synthetic and conventional MRI. Finally, the sample size needs to be further increased.

In conclusion, the rates of detection of sacroiliac joint lesions in axSpA achieved with synthetic MRI were similar to those of conventional MRI. Furthermore, quantitative mapping generated by synthetic MRI could accurately quantify BME and fat metaplasia. Therefore, synthetic MRI not only is as valuable as conventional MRI in the diagnosis of sacroiliac joint lesions in axSpA patients, but also enables us to obtain accurate, repeatable, and objective quantitative values that can provide more information about axSpA. If further longitudinal and correlation studies are carried out, synthetic MRI has the potential to act as an imaging quantitative index that reflects pathophysiological changes, which in turn could guide treatment and inform prognosis in patients with axSpA. Therefore, we believe that the prospects for the clinical application of synthetic MRI are good. However, a potential limitation of the synthetic MRI technique is the lack of stability and repeatability between different MR scanners and parameters. For better clinical application, multicenter prospective studies are needed to verify the stability and repeatability of the synthetic MRI technique using different MR scanners and scanning parameters.

## Data availability statement

The raw data supporting the conclusions of this article will be made available by the authors, without undue reservation.

## Ethics statement

This prospective study was approved by Ethics Committee of the Fifth Affiliated Hospital of Sun Yat-sen University, and written informed consent was obtained from all participants before the study.

## Author contributions

KZ: data analysis and writing the manuscript; CL: data collection and data analysis; YZ, WL, and XL: data collection; JZ and GH: revise the manuscript. All authors contributed to the article and approved the submitted version.

## Funding

The Science and Technology Project in the Social Development Field of Zhuhai City, Guangdong Province, China (No. ZH22036201210066PWC) and the Clinical IIT Research Project of the Fifth Affiliated Hospital of Sun Yat-sen University (No. YNZZ2020-06).

## Conflict of interest

The authors declare that the research was conducted in the absence of any commercial or financial relationships that could be construed as a potential conflict of interest.

## Publisher’s note

All claims expressed in this article are solely those of the authors and do not necessarily represent those of their affiliated organizations, or those of the publisher, the editors and the reviewers. Any product that may be evaluated in this article, or claim that may be made by its manufacturer, is not guaranteed or endorsed by the publisher.
